# Case Report: Acute methicillin-sensitive *Staphylococcus aureus* pericarditis in a diabetic patient

**DOI:** 10.3389/fcvm.2025.1674940

**Published:** 2025-11-14

**Authors:** Daniel J. Lim, Richard Lu, Edwin C. Y. Sng, Felix M. Uy, Wei L. Huang, Siang C. Chai, Anthony Yii, Ing X. Soo, Jenn N. Khoo, X. Ruan

**Affiliations:** 1 Department of Cardiology, Changi General Hospital, Singapore, Singapore; 2 Department of Infectious Diseases, Changi General Hospital, Singapore, Singapore; 3 Department of Respiratory and Critical Care Medicine, Changi General Hospital, Singapore, Singapore; 4 Department of Cardiothoracic Surgery, National Heart Centre Singapore, Singapore, Singapore; 5 Department of Diagnostic Radiology, Changi General Hospital, Singapore, Singapore

**Keywords:** bacterial pericarditis, methicillin-sensitive *Staphylococcus aureus* (MSSA), immune dysregulation, tuberculosis, multimodal imaging, pericardial window

## Abstract

**Introduction:**

Bacterial pericarditis is rare in the antibiotic era but remains potentially fatal due to rapid progression and high mortality. Herein, we report an unusual case of methicillin-sensitive *Staphylococcus aureus* (MSSA) pericarditis with a transudative pleural effusion in a patient with poorly controlled type 2 diabetes mellitus (DM), illustrating the diagnostic and therapeutic challenges in a complex patient.

**Patient concerns and clinical findings:**

A 47-year-old female patient with a history of presumptive ischemic cardiomyopathy, uncontrolled DM (a glycated hemoglobin level of 14.2%), and treated pulmonary tuberculosis (TB) presented with pleuritic chest tightness, fever, and dyspnea. Examinations and investigations revealed a moderate-to-large pericardial effusion, ST-segment elevation on an electrocardiogram, and high inflammatory markers. Imaging showed a pericardial effusion, raising suspicion for bacterial pericarditis. Pleural tap of an adjacent pleural effusion nearby was however transudative.

**Diagnosis, interventions, and outcomes:**

Pericardiocentesis was not feasible due to the loculated pericardial effusion and absence of a safe window; however, *S. aureus* was detected by polymerase chain reaction testing of the patient’s pleural fluid. Cardiothoracic surgeons performed a pericardial window and biopsy, confirming MSSA pericarditis. Surgical drainage was successful and the patient completed 6 weeks of intravenous cefazolin with full recovery.

**Conclusion:**

This case emphasizes the need to consider bacterial etiologies, including MSSA, when evaluating pericarditis in immunocompromised patients, especially those with DM or prior TB. Multimodal imaging, molecular diagnostics, and early surgical consultation are important in cases where pericardiocentesis is not feasible. Invasive diagnostic strategies may be critical for achieving a microbiological diagnosis and ensuring timely source control. Multidisciplinary collaboration is essential when managing complex pericardial infections to optimize diagnostic certainty and outcomes.

## Introduction

Bacterial pericarditis is rare but remains life-threatening in the antibiotic era (1). Mortality historically approached 100% in untreated cases and remains as high as 40%, even with appropriate drainage and antibiotics (2). While *Staphylococcus aureus* is a recognized etiology, manifestations due to methicillin-sensitive *S. aureus* (MSSA) are uncommon, especially in immunocompetent patients. Multiple case series have documented that nearly all patients present with a pericardial effusion, the majority develop tamponade, and a substantial minority exhibit loculated effusions or require surgical intervention (3, 4).

We report an unusual case of MSSA pericarditis in a patient with poorly controlled type 2 diabetes mellitus (DM) and a history of treated tuberculosis (TB), complicated by a loculated pericardial effusion without a safe window for pericardiocentesis. Recent reports have described MSSA pericarditis in post-COVID-19 immunocompetent hosts and in diabetic patients with septic bacteremia, typically presenting with tamponade physiology and managed via pericardiocentesis (5, 6). In contrast, our case highlights the additional complexity posed by prior TB and a surgically managed loculated effusion, underscoring the role of multimodal imaging, multidisciplinary management, and timely surgical intervention in confirming a microbiological diagnosis and achieving source control.

## Case description

The patient was a 47-year-old woman who presented with a 4-day history of intermittent, exertional, pleuritic chest tightness and shortness of breath that was exacerbated by lying down and coughing and improved when sitting up. She had a 5-day history of fever (Tmax 38.3°C) with no localizing symptoms, constitutional symptoms, or night sweats.

Her past medical history included presumptive ischemic cardiomyopathy, with a left ventricular ejection fraction (LVEF) of 40% and regional wall motion abnormalities, which had not been further evaluated. Other pertinent medical history included poorly controlled type 2 DM [a glycated hemoglobin (HbA1c) level of 14.2%] and drug-susceptible pulmonary TB, for which she had completed 9 months of first-line TB treatment, consisting of rifampicin, isoniazid, pyrazinamide, and ethambutol, via directly observed therapy (DOT) 3 years prior.

On examination, she was febrile at 38.4°C, with a blood pressure of 118/79 mmHg, a heart rate of 104 beats per minute, and oxygen saturation of 98% on room air. She had dual heart sounds (S1 and S2) with no murmur or pericardial rub. Bilateral coarse crepitations were heard on auscultation, which were worse on the left, with no pedal edema. A bedside transthoracic echocardiogram (TTE) showed a pericardial effusion that was largest in size (2.1 cm) anterior to the right ventricle (RV) and smallest (∼0.2 cm) at the apex. The pericardial effusion was best viewed in the subcostal window, with no significant valvulopathy or regional wall motion abnormality (RWMA). Her admission 12-lead electrocardiogram (ECG) showed sinus rhythm with ST-segment elevation in the anterior precordial leads that was maximal in V2-V3, with reciprocal ST-segment depression and PR-segment elevation in lead augmented Voltage Right arm (aVR) (Supplementary Figure S1A); a repeat ECG tracing 20 min later (Supplementary Figure S1B) demonstrated no dynamic ST-T changes. The high-sensitivity troponin T results trended as follows: 8 → 9 → 7 ng/L. Furthermore, the patient’s NT-proBNP level was 332.0 pg/mL. The laboratory test results showed elevated inflammatory markers, with the full blood count showing leukocytosis at 17.3 × 10^3^/μL, neutrophils at 14.8 × 10^3^/μL (85.2%), lymphocytes at 1.1 × 10^3^/μL (6.4%) and monocytes at 1.4 × 10^3^/μL (7.9%), serum C-reactive protein at 272.7 mg/L, and serum procalcitonin at 0.66 μg/L. A chest x-ray (CXR) showed stable post-TB changes without new consolidation.

The patient was empirically treated with intravenous (IV) piperacillin/tazobactam for the sepsis and aspirin and colchicine for acute pericarditis. There was concomitant diabetic ketoacidosis (DKA) precipitated by the sepsis, as evidenced by a serum bicarbonate level of 13 mmol/L, serum glucose of 30.9 mmol/L and serum ketones of 1.4 mmol/L, and a venous blood gas conducted on admission showing a pH of 7.37, pCO_2_ of 36.3 mmHg, and serum bicarbonate of 18.0 mmol/L, which required 3 days of treatment with IV insulin. A formal TTE showed a moderate to large loculated pericardial effusion with septations and no safe window for pericardiocentesis (Supplementary Figure S3, Video S1). Despite there being RV diastolic collapse (Supplementary Video S2), there were no clinical symptoms of tamponade; thus, pericardiocentesis was not pursued.

**Figure 1:**
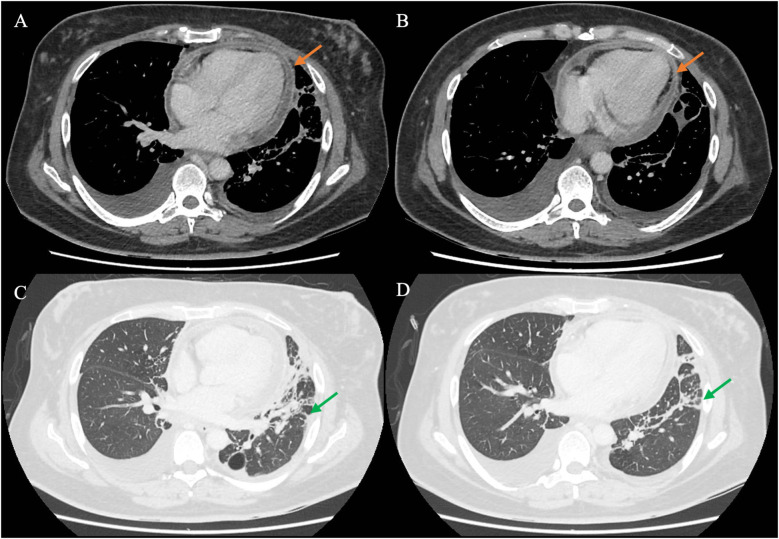
CT of the chest of the patient using different windows. (**A**,**B**) The chest CT in the soft tissue/mediastinal window, highlighting the pericardium (orange arrow) surrounding the heart. The findings are consistent with acute pericarditis with an associated pericardial effusion. (**C**,**D**) The lung window, in which streaky consolidation in the left lung is more clearly visualized (green arrow). CT, computed tomography.

**Figure 2:**
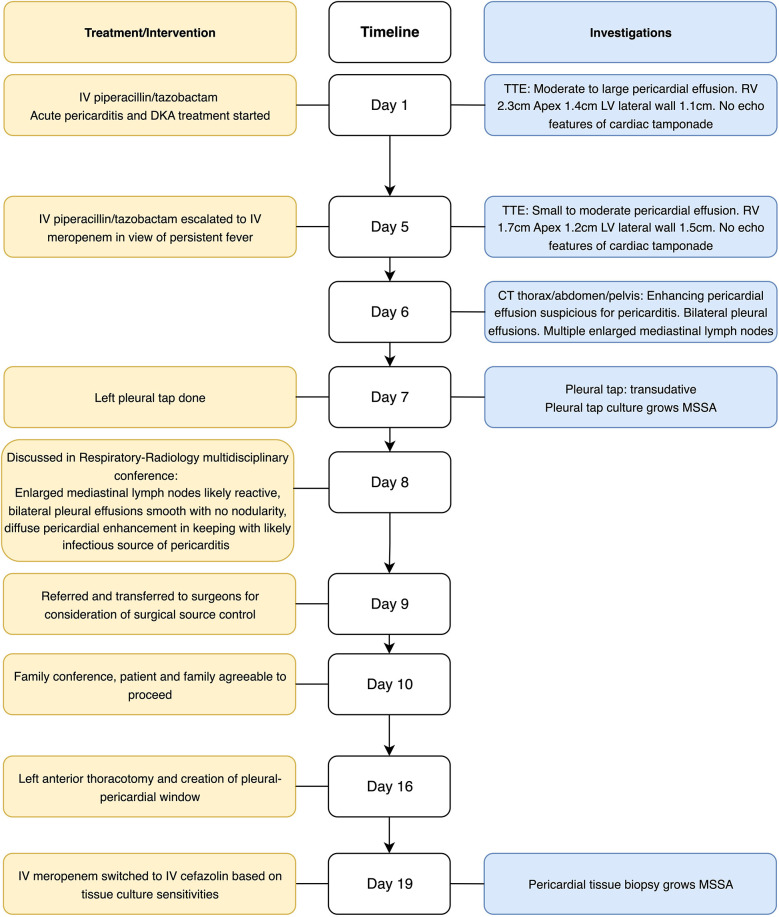
Timeline of admission. CT, computed tomography; DKA, diabetic ketoacidosis; IV, intravenous; MSSA, methicillin-sensitive *S. aureus*; TTE, transthoracic echocardiogram; RV, right ventricle; LV, left ventricle.

As the patient’s high fever did not abate on IV piperacillin/tazobactam, contrast-enhanced computed tomography (CT) of the thorax, abdomen, and pelvis was performed, revealing patchy consolidation in both lung lobes with air bronchograms in the right upper lobe and lingula segment (Supplementary Figure S2) and areas of bronchiectasis, scarring, and mediastinal lymphadenopathy that was stable compared to a previous examination conducted several years ago. These findings were consistent with post-tuberculosis lung disease. There were small bilateral pleural effusions. In addition, CT indicated an enhanced pericardial effusion 1.4 cm in size, consistent with pericarditis (Figure 1). The Department of Infectious Diseases was consulted, who recommended initiating IV meropenem, which treats the common etiologies of bacterial pericarditis and melioidosis, for which she was at risk due to her poorly controlled diabetes (Figure 2).

## Differential diagnosis

The initial differentials were bacterial pericarditis due to pyogenic bacteria, including *S. aureus*, *Streptococcus pneumoniae*, and *Burkholderia pseudomallei* (the agent that causes melioidosis). Tuberculous pericarditis was considered but deemed less likely as she had completed TB treatment.

## Investigation

The patient underwent a left diagnostic thoracocentesis by respiratory physician from the Department of Respiratory Medicine based on the clinical suspicion that the pleural effusions could have been secondarily infected by the pericardium. Pleural biochemistry revealed a neutrophilic transudate, and nucleic acid amplification testing (NAAT) was positive for *S. aureus*. The pleural fluid culture was negative (Table 1A). Despite 9 days of broad-spectrum IV antibiotics, she remained febrile. As there was no safe window for pericardiocentesis, the Department of Cardiothoracic Surgery was consulted for the creation of a pleural–pericardial window and drainage of the pericardial fluid. This procedure served both diagnostic purposes—to ascertain whether the positive *S. aureus* polymerase chain reaction (PCR) from the pleural fluid represented a true infection, given the negative fluid culture—and therapeutic purposes, as the patient had persistent fever and sepsis despite appropriate antibiotic therapy, necessitating definitive source control. The patient underwent a left anterior thoracotomy and creation of a pleural–pericardial window on day 16 of admission (Figure 2).

**Table 1:** Summary of the molecular diagnostics.

A. Investigations of pleural and serum fluid from the pleural tap			
Investigation	Pleural fluid	Serum	Ratio
Total protein (g/L)	24.0	79.0	<0.5
Lactate dehydrogenase (U/L)	125	273 (90–190)	<0.6
Albumin (g/L)	11	25	
Glucose (mmol/L)	12.9	16.8	
Cell count (cells/mm^3^)	594		
Neutrophils (%)	80		
Lymphocyte (%)	20		
pH	>7.80		
Adenosine deaminase (U/L)	7.7		
B. Microbiological investigations			
Investigation	Finding		
Blood cultures	No bacterial growth in aerobic and anaerobic blood cultures × 3 sets		
Urine cultures	No bacterial growth × 1 set		
Pleural fluid	Bacterial PCR: *S. aureus*		
	*B. pseudomallei* PCR: not detected		
	Gram stain and aerobic culture: No bacterial growth		
	TB DNA PCR: *M. tuberculosis* not detected		
	AFB smear and culture: no acid-fast bacilli seen		
	Fungal culture: no growth		
Pericardial tissue and fluid	Tissue aerobic culture: Methicillin-sensitive *S. aureus*		
	Tissue anaerobic culture: no bacterial growth		
	Fluid bacterial PCR: *S. aureus*		
	Pneumonia multiplex (*Chlamydia pneumoniae*, *Mycobacterium pneumoniae, Legionella pneumophila*, and *Bordetella pertussis*) PCR: not detected		
	*B. pseudomallei* PCR: not detected		
	Fungal culture: no growth		
Sputum	TB DNA PCR: *M. tuberculosis* not detected		

AFB, acid-fast bacilli; DNA, deoxyribonucleic acid; TB, tuberculosis; PCR, polymerase chain reaction.

The intraoperative findings noted extensive adhesions with multiple pleural loculations and a serous pleural effusion. The pericardium was thickened and inflamed with minimal pericardial fluid or purulent material. The myocardial surface was also inflamed with dense adhesions within the pericardial cavity. A small section of the pericardium was excised to create the window, with specimens of pleural effusion and pericardium sent for histopathological culture and further analysis (Table 1B). The intraoperative tissue culture and bacterial PCR of the pericardial fluid both grew MSSA. Following surgery, the patient’s clinical status improved. The patient was discharged well and completed 6 weeks of IV cefazolin for MSSA pericarditis in an outpatient setting, with a full clinical recovery. A follow-up TTE 3 months after the completion of IV cefazolin showed that the pericardial effusion remained small, 2 mm at most adjacent to the RV, with the patient returning to her baseline health.

## Discussion

We report a case of acute pericarditis caused by MSSA. *S. aureus* is a recognized cause of bacterial pericarditis. However, with the advent of antibiotics, bacterial pericarditis is now seldom encountered in clinical practice (1). While viral and tuberculous pericarditis are currently the most common, bacterial pericarditis should still be considered, especially in the presence of risk factors (1). MSSA-related pericarditis typically arises in immunocompromised individuals or those with predisposing conditions, such as DM, malignancy, or prior cardiothoracic interventions (1, 7). The reported risk factors comprise a pre-existing pericardial effusion, immunosuppression, chronic disease (alcohol misuse or uremia), cardiac surgery, and chest trauma (7). In this case, we postulate that the patient's immunocompromised state due to poorly controlled DM predisposed her to *S. aureus* infection. Immune dysregulation in DM is associated with impaired innate and adaptive immune responses, resulting in delayed bacterial clearance (8). The pericardium was likely to have been hematogenously seeded during an episode of bacteremia. Other routes of infection include direct spread from an intrathoracic focus of infection (e.g., pneumonia or pleural empyema), extension from a myocardial focus, or direct inoculation after trauma or thoracic/cardiac surgery; infections are rarely due to a primary bacterial infection of the pericardium (9).

If pericarditis is suspected, a TTE is recommended to identify and quantify any pericardial effusions and assess for cardiac tamponade, which has been reported in previous cases of staphylococcal pericarditis (10). Treatment of bacterial pericarditis requires both effective antibiotics and source control (11). Despite the presence of early RV diastolic collapse on the initial TTE, given that our patient displayed no clinical symptoms of tamponade and there was no safe window, pericardiocentesis was not performed, and the Department of Cardiothoracic Surgery was promptly consulted.

TB and melioidosis were the differentials considered. TB remains endemic with non-specific symptoms; a definitive diagnosis requires detecting *Mycobacterium tuberculosis* in pericardial fluid or tissue by culture or nucleic acid testing (12). Treatment for TB has been posited to have an immunosuppressive effect; our patient, having completed DOT, may therefore have been at elevated risk for MSSA infection (13). Melioidosis, caused by *B. pseudomallei* from soil or water exposure, ranges from localized disease to fulminant sepsis; pericarditis is rare but has been reported (14, 15). Individuals with diabetes, chronic kidney disease, or immunosuppression are at higher risk; a diagnosis is made by culture and treatment comprises intensive and eradication phases (14).

MSSA and methicillin-resistant *S. aureus* (MRSA) pericarditis present similarly, but the risk profiles and management differ. MSSA pericarditis is often community-acquired and linked to contiguous or hematogenous spread from foci such as pneumonia, mediastinitis, endocarditis, or bacteremia, particularly in patients with systemic illness or immunosuppression (16). Our patient, therefore, presented atypically, given the subacute course with no clear source and minimal purulence. By contrast, MRSA pericarditis more commonly follows healthcare exposure or procedures. While they share risk factors such as immunosuppression and pericardial injury, tamponade is common in MRSA pericarditis (17). For treatment, definitive therapy should target susceptibilities: anti-staphylococcal β-lactams are preferred for MSSA, whereas MRSA requires agents such as vancomycin or daptomycin; in all cases, early pericardial drainage or a surgical window is key, and the typical antibiotic treatment duration is 4–6 weeks. The mortality rate remains substantial, with purulent pericarditis resulting in a mortality rate of ∼40% despite treatment, while a systematic review of MRSA pericarditis reported a 20% mortality rate, with tamponade in 83% of cases (3).

Fungal pericarditis was another important differential considered. Differentiating fungal from bacterial pericarditis was crucial as the etiology determines the risk, therapy, and outcomes. Fungal pericarditis, commonly due to *Candida* spp. and rarely due to *Aspergillus* spp., occurs predominantly in immunocompromised or post-operative hosts (18). Risk factors comprise old age, diabetes, immunosuppression due to malignancy, prolonged steroid therapy, and septicemia, and immediate treatment with amphotericin B or an echinocandin with surgical drainage is mandated (19). Fungal pericarditis can proceed to cardiac tamponade and pericardial constriction, which results in a high mortality rate approaching ∼50%, particularly when appropriate antifungal therapy is delayed (19, 20), thus necessitating expedient detection and diagnosis. In our patient, fungal cultures of pleural and pericardial fluid yielded no fungal growth (Table 1B).

Three sets of aerobic/anaerobic blood cultures were obtained and were negative (Table 1B); this is consistent with a contemporary series in which only ∼50%–60% of purulent pericarditis cases had positive blood cultures (3, 21). Because blood culture sensitivity is limited and prior antibiotics can further reduce the yield, a definitive diagnosis relies on pericardial fluid/tissue cultures and molecular assays; in our case, the surgical window enabled PCR of pericardial tissue culture, which confirmed MSSA.

CT provided additional insights, not only revealing pericardial enhancement (Figure 1) but additionally identifying small pleural effusions not evident on chest radiograph that provided an alternative target for obtaining diagnostic specimens. As *S. aureus,* a known cause of bacterial pericarditis, was detected in nucleic acid amplification testing of the pleural effusion, the suspicion for *S. aureus* pericarditis was heightened, prompting the decision to conduct a pericardial window and drain the pericardial fluid.

The complexity of this case necessitated a multidisciplinary approach, integrating expertise from the departments of Cardiology, Infectious Diseases, Respiratory Medicine, Radiology, and Cardiothoracic Surgery. Given the diagnostic uncertainty and lack of a safe pericardiocentesis approach, collaboration between these specialties led to the decision to pursue a timely pericardial window and biopsy, ultimately yielding a definitive microbiological diagnosis of MSSA pericarditis before further clinical deterioration occurred. The role of the Department of Cardiothoracic Surgery was pivotal, as the pericardial window facilitated tissue sampling for microbiological diagnosis and achieved source control. The patient’s persistent fever despite 9 days of broad-spectrum β-lactams suggested inadequate source control of a loculated infection; such effusions often require surgical drainage (17). Piperacillin/tazobactam may underperform in high-burden MSSA due to an inoculum effect (22), so we completed 6 weeks of targeted cefazolin after the window, guided by the Infectious Diseases team, with resolution of the fever. The coordinated management strategy exemplifies the importance of team-based care when addressing challenging infectious and cardiac conditions. Unlike other recent reports of MSSA pericarditis occurring in post-COVID immunocompetent or diabetic patients with tamponade physiology (5, 6), our patient's risk profile and effusion characteristics necessitated a surgical pericardial window rather than pericardiocentesis, underscoring the need for individualized management strategies.

## Conclusions

Our case of MSSA pericarditis reflects the importance of maintaining a broad differential diagnostic framework, even when the clinical diagnosis of acute pericarditis is evident. While viral pericarditis is most common, bacterial etiologies should be suspected in patients, especially in those with risk factors. This case highlights the crucial role of multimodal imaging, including echocardiography and CT, in characterizing pericardial effusions, ruling out differentials and guiding management decisions. When the microbiological diagnosis is unknown, pursuing invasive diagnostics, such as a pericardial biopsy and a pericardial window, can provide a definitive diagnosis and achieve source control. Multidisciplinary management is essential in ensuring timely diagnostics and therapeutic intervention in complex cases with pericardial diseases.

## Data Availability

The data that support the findings of this study will be available from the corresponding author upon reasonable request.

## Supplementary material

Supplementary Video S1 Subcostal view of the TTE on admission demonstrating the loculated effusion with the liver confounding the subcostal approach for pericardiocentesis.

Supplementary Video S2 Parasternal long axis TTE on admission demonstrating diastolic right ventricular collapse.

Supplementary Figure S1 Electrocardiogram of the patient on admission. (**A**) Electrocardiogram conducted on admission. (**B**) Electrocardiogram conducted 20 min after admission, displaying no dynamic changes in ST-T segments. Supplementary Figure S2 Computed tomography of the thorax showing air bronchograms and consolidation in the right upper lobe (red arrow). Supplementary Figure S3 Apical 4-chamber view of the pericardial effusion on a transthoracic echocardiogram showing no safe window for pericardiocentesis. Supplementary Figure S4 The patient's chronic medication list. The patient's chronic medications comprise the following: PO amlodipine 5 mg OM, PO enalapril 5 mg BD, PO linagliptin 5 mg OM, and SC Lantus (insulin glargine) SoloStar 24 units at bedtime.
