# Supplementary material for: Case Report: Acute methicillin-sensitive Staphylococcus aureus pericarditis in a diabetic patient
Source: Front Cardiovasc Med. 2025 Nov 14;12:1674940. doi: 10.3389/fcvm.2025.1674940 (PMC12660183; doi:10.3389/fcvm.2025.1674940)
Supplement: Supplementary Figure S1 — Electrocardiogram of the patient on admission. (A) Electrocardiogram conducted on admission. (B) Electrocardiogram conducted 20 min after admission, displaying no dynamic changes in ST-T segments. Supplementary Figure S2 Computed tomography of the thorax showing air bronchograms and consolidation in the right upper lobe (red arrow). Supplementary Figure S3 Apical 4-chamber view of the pericardial effusion on a transthoracic echocardiogram showing no safe window for pericardiocentesis. Supplementary Figure S4 The patient's chronic medication list. The patient's chronic medications comprise the following: PO amlodipine 5 mg OM, PO enalapril 5 mg BD, PO linagliptin 5 mg OM, and SC Lantus (insulin glargine) SoloStar 24 units at bedtime. [file Datasheet1.docx]

**SUPPLEMENTARY FILES**

**Supplementary Figure 1. Electrocardiogram of the patient on admission**

B

A


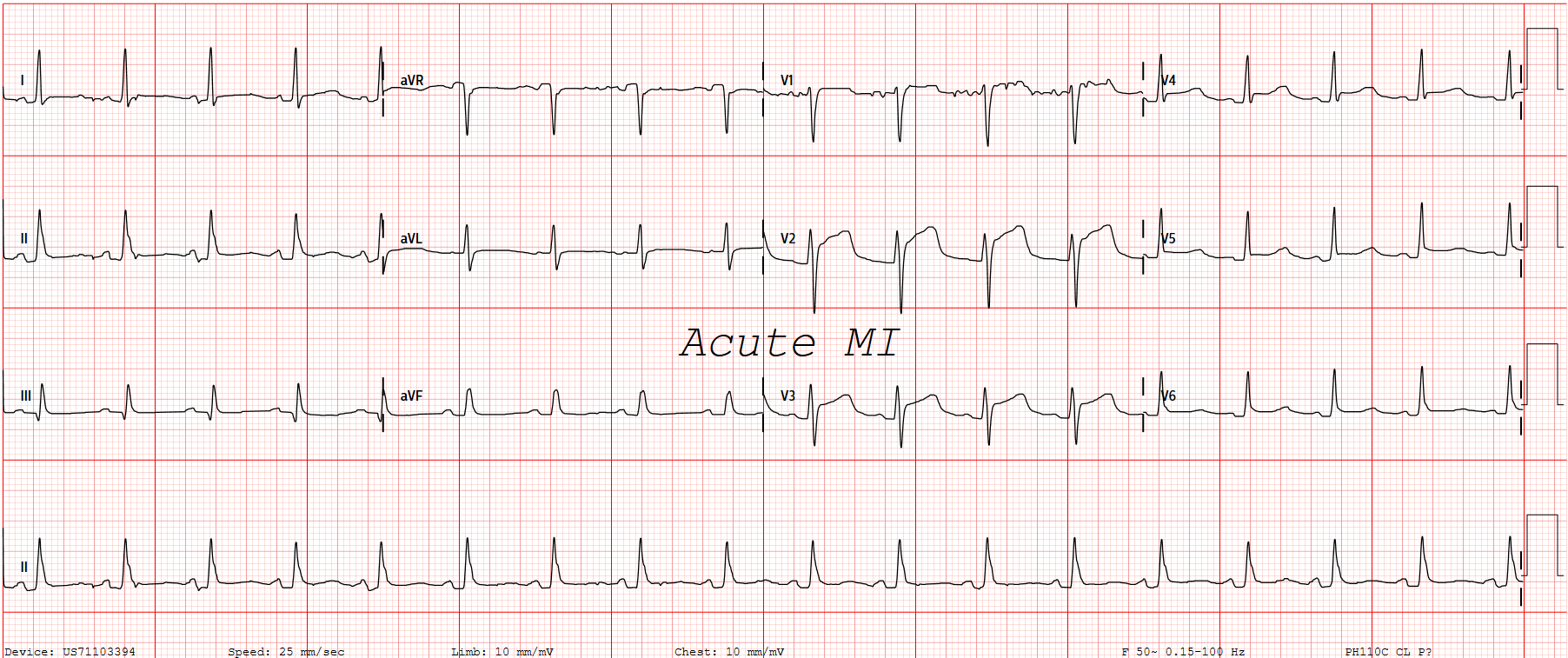

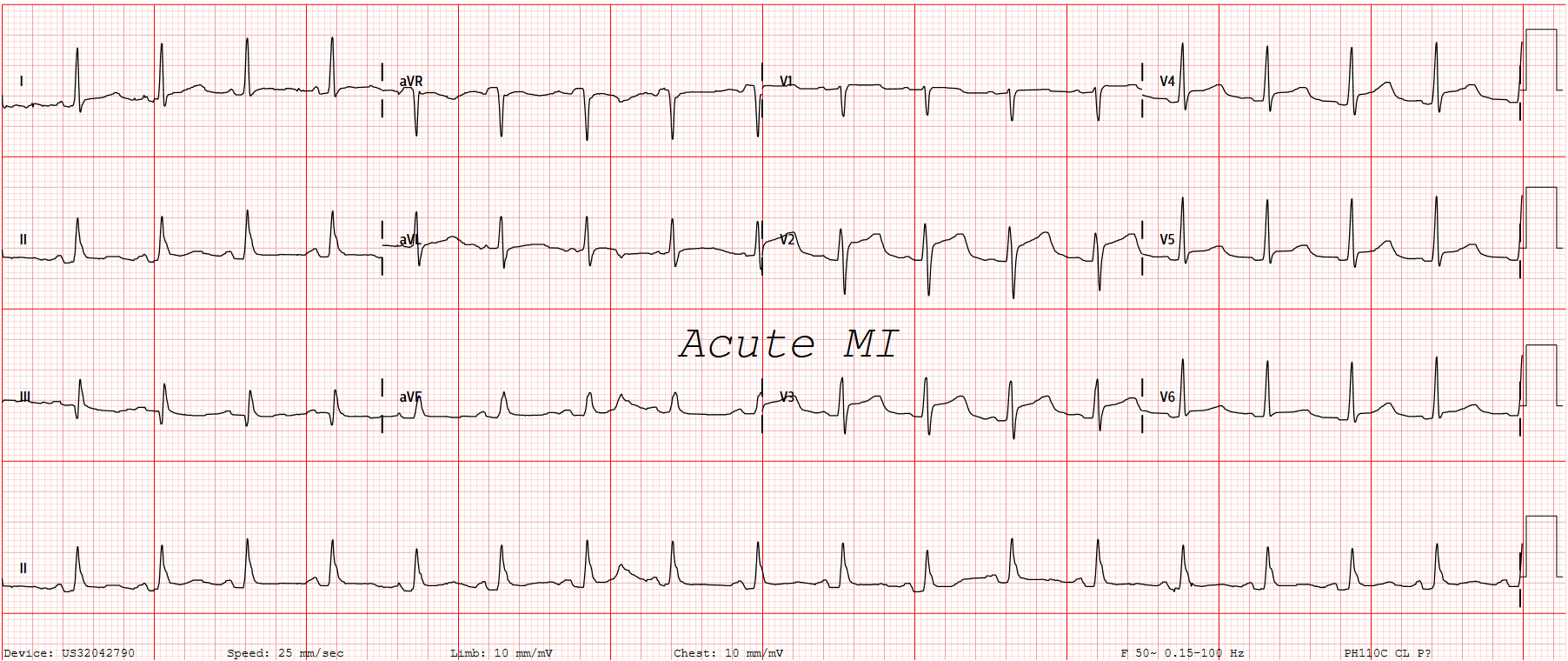


(A): electrocardiogram done on admission; (B): electrocardiogram done 20 minutes after, displaying no dynamic changes of ST-T segments

**Supplementary Figure 2. Computed tomography of the thorax showing air bronchograms and consolidation in the right upper lobe (red arrow)**


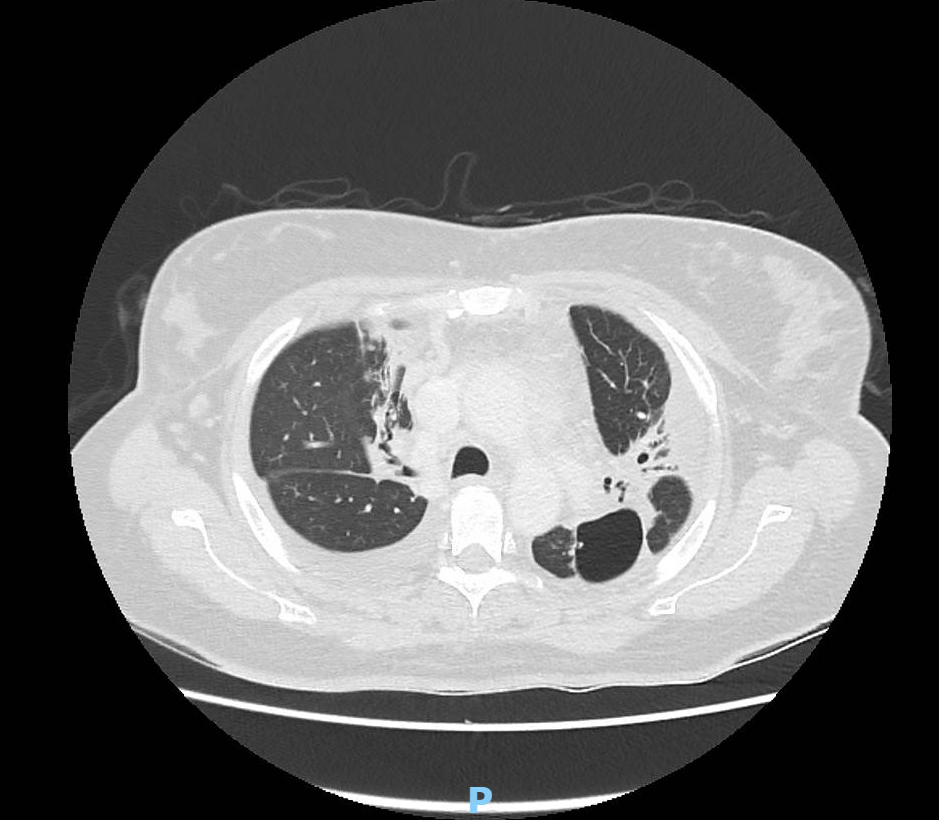


**Supplementary Figure 3. Apical 4-chamber view of the pericardial effusion on transthoracic echocardiogram showing no safe window for pericardiocentesis.**

**
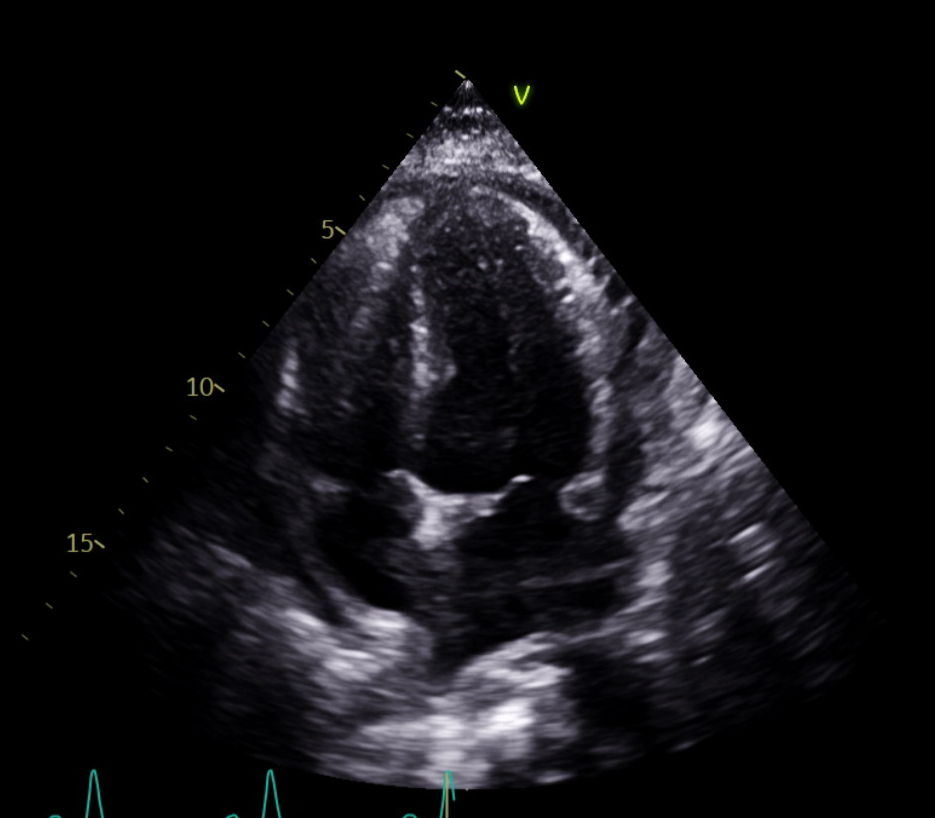
**

**Supplementary File 4. Patient’s chronic medication list**

The patient’s chronic medications comprise: PO amlodipine 5mg OM, PO enalapril 5mg BD, PO linagliptin 5mg OM, SC lantus (insulin glargine) solostar 24units at bedtime
